# Sublethal toxicities of 2,4-dinitrophenol as inferred from online self-reports

**DOI:** 10.1371/journal.pone.0290630

**Published:** 2023-09-13

**Authors:** Ali Abdelati, Michele M. Burns, Michael Chary

**Affiliations:** 1 Weill Cornell Medicine-Qatar, Ar-rayan, Qatar; 2 Division of Emergency Medicine, Harvard Medical Toxicology Fellowship, Boston Children’s Hospital, Boston, MA, United States of America; 3 Division of Medical Toxicology, Department of Emergency Medicine, Weill Cornell Medical College, NY, NY, United States of America; 4 Department of Emergency Medicine, New York Presbyterian Queens, Flushing, New York, United States of America; University of Wisconsin–Madison, UNITED STATES

## Abstract

**Introduction:**

2,4-dinitrophenol (DNP) is a mitochondrial toxin sometimes used as a weight loss agent. Reports of fatalities from DNP have been increasing since 2000, suggesting an increase in use. Our understanding of DNP toxicity in humans comes from reports to Poison Control and postmortem analyses, sources that are biased to more extreme presentations. This leads to a gap in our knowledge about the adverse effects of DNP at nonlethal doses. Here we investigate the doses and effects of DNP as reported online.

**Methods:**

We analyzed publicly available Internet posts that we collected from 2017–2019. The posts came from anonymous users or users who voluntarily self-identified. We collected data from websites whose terms of use allow for the secondary analysis of data that their users agree to make public. We used natural language processing techniques that we had previously developed to extract doses, effects, and substances mentioned in each post.

**Results:**

We collected 1,630 posts across 5 online forums and the Reddit forum r/DNP. The posts were from 1,234 unique usernames. The most commonly reported doses were between 150 to 300 mg each day followed by 300 to 450 mg each day. At those doses, the most reported adverse effects were profuse sweating and fatigue. Reports of thermoregulatory (sweating, feeling hot flashes or flushed), fatigue-related, and neurologically related symptoms were statistically significantly more frequent at reported daily doses greater than 150 mg than doses below 150 mg (post-hoc χ^2^-test with Bonferroni correction). The effects were judged as plausible by two board-certified medical toxicologists. Triiodothyronine, clenbuterol, testosterone, and trenbolone, an androgenic anabolic steroid were the most significantly co-mentioned substances.

**Conclusions:**

Fatigue, increased body temperature, and paresthesias from DNP are reported more frequently at doses greater than 150 mg each day than at doses less than 150 mg each day. Online discussions of DNP frequently mention androgenic anabolic steroids and other weight loss agents.

## Introduction

2,4-dinitrophenol (DNP) uncouples oxidative phosphorylation, dissipating the proton gradient by creating holes in the inner mitochondrial membrane [1]. DNP use is associated with life-threatening hyperthermia, cataracts, and cardiovascular collapse [2]. It was first prescribed by physicians for weight loss in 1933 [3, 4]. In 1938, the FDA declared DNP as not fit for human consumption after reports of fatal hyperthermia and progressive cataracts [5], leading to its removal from the market that same year. It is currently approved in the United States only as an industrial pesticide.

Despite the ban by the FDA and its toxicity, DNP use seems to be increasing. Of the 25 reports of fatalities to US Poison Control Centers, 12 occurred after 2000 [2]. Of those 12 reports, 7 involved people under 25 taking DNP to reduce body fat without losing muscle mass. Across the world reported calls annually to Poison Control increased from 4 in 2010 to 71 in 2015 to 53 in 2019 [6]. Historically associated with self-identifying men, use among those who identify as women may be increasing [7].

DNP can be an alluring option to those trying to rapidly lose weight or decrease body fat. Anecdotal reports hint that using DNP can lead to 3–5 kg of weight loss each week [8]. In contrast, the 5 medications the Food and Drug Administration (FDA) has approved lead to 3–5 kg weight loss after one year [9]. These medications are orlistat (a lipase inhibitor) [10], phentermine (a stimulant)/topiramate (an anticonvulsant), lorcaserin (a central serotonin 2C agonist), naltrexone (an opioid receptor antagonist)/bupropion (a stimulant), liraglutide (a glucagon-like peptide-1 agonist) [11] and, most recently, semaglutide. Lorcaserin was voluntarily withdrawn because of a possible increased risk of cancer [12].

Our understanding of DNP toxicity comes primarily from postmortem analyses and reports from Poison Control, leading to a gap in our knowledge about usage and sublethal effects. Social media provide an informal registry of behavior. This registry can be valuable for understanding substance use that is stigmatized or illicit. People discuss DNP use online [13, 14]. A content analysis of 14 users from one bodybuilding discussion forum found that those users were aware of the toxicity of DNP, but still chose to use it to “cut” (reduce body fat) [15, 16].

In addition to thematic analyses of behavior, social media may provide information traditionally gathered from controlled experimentation, such as dose-effect relationships. We previously demonstrated that natural language processing of narrative text from social media can identify dose-effect relationships [17] and patterns of combining substances [18]. The purpose of this study is to investigate the dose-effect associations of 2,4-dinitrophenol (DNP) as reported on social media in order to better understand the effects of DNP at nonlethal doses.

## Methods

**Fig 1:**
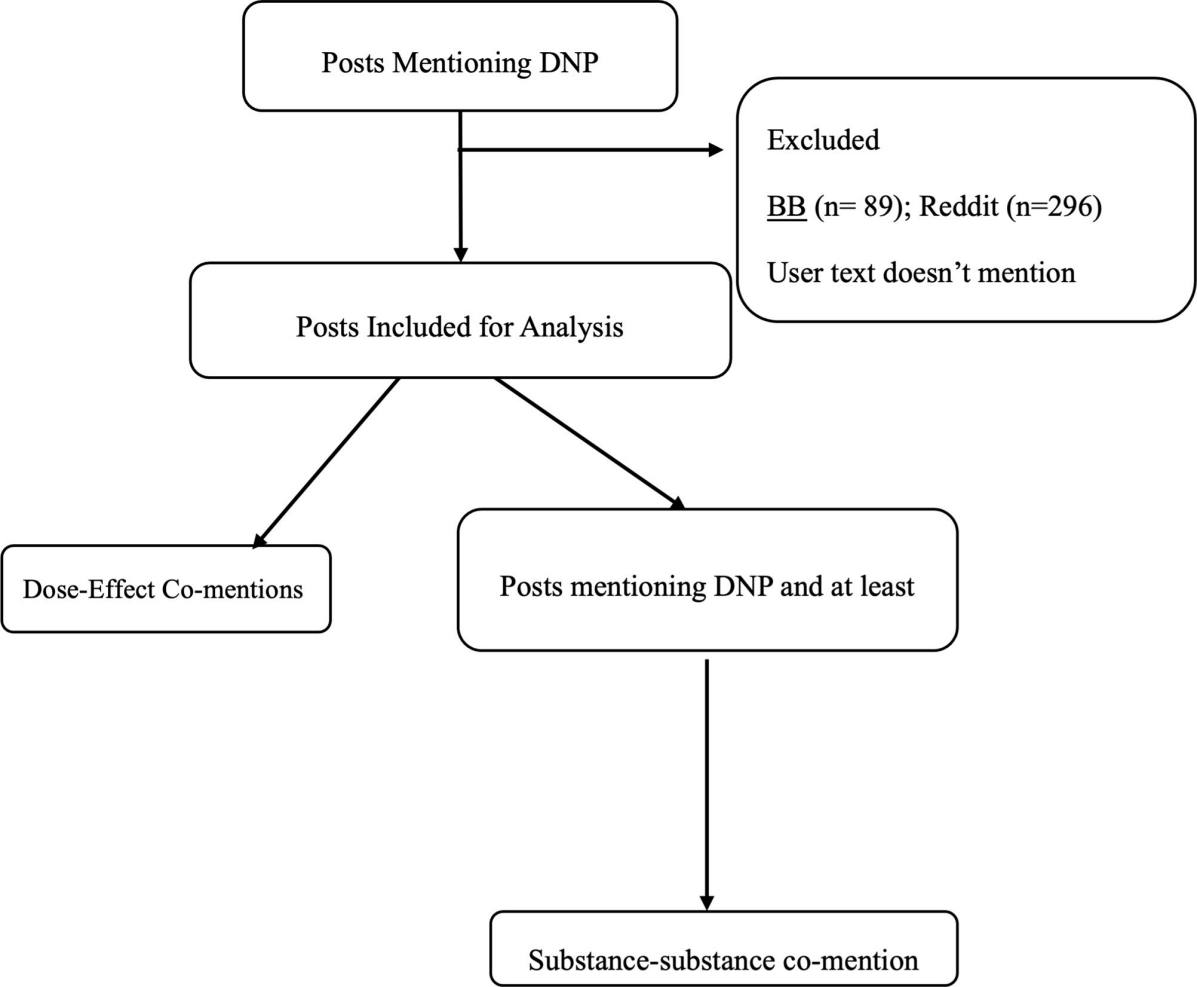
Schematic of study flow. DNP, 2,4-dinitrophenol. BB, online bulletin boards also called discussion forums.

We extracted posts that mentioned DNP from 5 online forums and the subreddit r/DNP. We excluded duplicate posts and posts selling DNP (Fig 1). On the subset of posts that also mentioned other substances, we calculated the correlation between the pattern of co-mention of those substances and DNP. From the posts mentioning only DNP, we calculated how often each dose was mentioned and the effects mentioned at each dose. All software was written in Python by author MC and is freely available at the GitHub repository dnp_ner_public and on request. In this section we use lowercase italic text refers to a specific Python library, *e*.*g*., *scipy*.

### Acquisition of data

We scraped publicly available posts that contained lexical variants of DNP (2,4-dinitrophenol, DNP, dinitrophenol, and all orthographic variants [*e*.*g*., DnP, Dnp, DNp]) from the websites listed in **Table 1**. For each post we retrieved the web page expressed in hypertext markup language (HTML) and used *scrapy* [19] to convert the HTML code into plain text. We chose websites analyzed in previous studies whose terms of service did not prohibit programmatic access of their content [13]. The posts are available online perpetually unless a discussion moderator removes them, or the web site shuts down. For reddit, we used *praw* to access the text of all posts and comments in r/DNP [20]. The corresponding author’s Institutional Review Board evaluated this project as a secondary analysis of publicly available data and not human subjects research (protocol number #23–01025586). This study involved no patient contact.

**Table 1:** Left column: Name of web site. Middle column: Number of posts from website that mention a DNP keyword. Right column: Median (interquartile range) of tokens (words, abbreviations, acronyms, see text for further explanation) in each post.

Website	No. of Posts	Tokens per post
IronDen	48	15 (5–34)
Steroidology	128	48 (20–110)
UGBodyBuilding	213	42 (18–88)
ThinkSteroids	64	55 (26–111)
Bodybuilding.com	208	28 (12–61)
Reddit	673	36 (14–73)

### Identification of mentions of DNP and coingestants

We used *nltk* [21] to label each word in each post by its most likely part of speech (*e*.*g*. noun, adjective). We manually reviewed words that were tagged as nouns or abbreviations to identify mentions of substances and map all synonyms and variations in spelling of each substance to one standard label. For example, we labelled posts that mention “Tren” or “trenbalone” as referring to trenbolone, an anabolic steroid and a derivative of 19-nortestosterone. Two authors reviewed the potential mentions of substances. The third was to adjudicate any disagreements standardizing words, but there were no disagreements.

We calculated the frequency of each standardized word as the combined frequency of all spelling variants and synonyms. For example, the frequency of “trenbolone” would be the combined frequency of “Tren” and “trenbalone” and any other mentions.

### Identification of doses

We used *nltk* to identify and extract numbers, whether written with numerals or written out, from each post. We then used *word2number* to convert numbers written in text into numerals, e.g., to convert three to 3. One author manually converted all doses to total daily dose. When determining dose-effect associations, we considered posts that mentioned only DNP and only one dose to avoid ambiguity.

### Association between mentions of DNP and other substances

To determine whether a substance was mentioned more with DNP than would be expected by chance we used the χ^2^-test. For each substance, we used the fraction of posts in which the substance occurred as the expected value and the fraction of posts in which the substance and DNP occurred as the observed value. We used the Benjamini-Hochberg correction to set the false discovery rate to 0.05 [22].

### Statistical significance of effects

We calculated the statistical significance of the association between the dose and number of times an effect was reported by using the χ^2^-test on the contingency table formed between reported doses (< 150 mg, 150 to 300 mg, 300 to 450 mg) and type of effect (fatigue, hydration, neurologic, dermatologic, or thermoregulatory). We investigated pairwise associations (*e*.*g*., between frequencies of effects at 150 to 300 mg and 300 to 450 mg) with the *post hoc* approach of pairwise χ^2^-tests. For the *post hoc* tests we adjusted the threshold for statistical significance by a Bonferroni correction factor of 6 (*i*.*e*., 3! Pairwise comparisons).

### Creation of effect classes

We identified by manual curation 111 user descriptions of symptoms. Based on their expert knowledge, authors MC and MMB, who are both board-certified and actively practicing medical toxicologists, created a taxonomy to map these user descriptions to 55 UMLS standardized terms. We further grouped those 55 UMLS standardized terms (*e*.*g*., fatigue, malaise) into 20 groups related to a physiologic function or dysregulation (*e*.*g*., grouping fatigue and malaise under *fatigue issue*). The mappings from user description to UMLS standardized terms and from UMLS standardized terms to groupings of functionally related terms are available at the GitHub repository.

### Validation

Two board-certified medical toxicologists adjudicated the effects and dosages mentioned as plausible or not plausible. There is no gold standard other than expert opinion available for the effects of DNP at the lower doses this manuscript studies. That expert opinion is based of extrapolation from effects at higher doses and reasoning from mitochondrial physiology.

## Results

We retrieved 661 unique posts (561 unique usernames) from 5 web forums and 969 posts (673 unique usernames) from the Reddit forum r/DNP posted between Dec 2017 and Dec 2018. (**1**). In the web forums, 583 (88.2%) mentioned DNP doses and effects and 140 (21.2%) mentioned DNP and an additional substance. Those 140 posts mentioned, in total, 67 additional substances. In r/DNP, 68 (10%) mentioned DNP doses and effects and 266 (40%) mentioned DNP and an additional substance.

### Dosage

**Fig 2:**
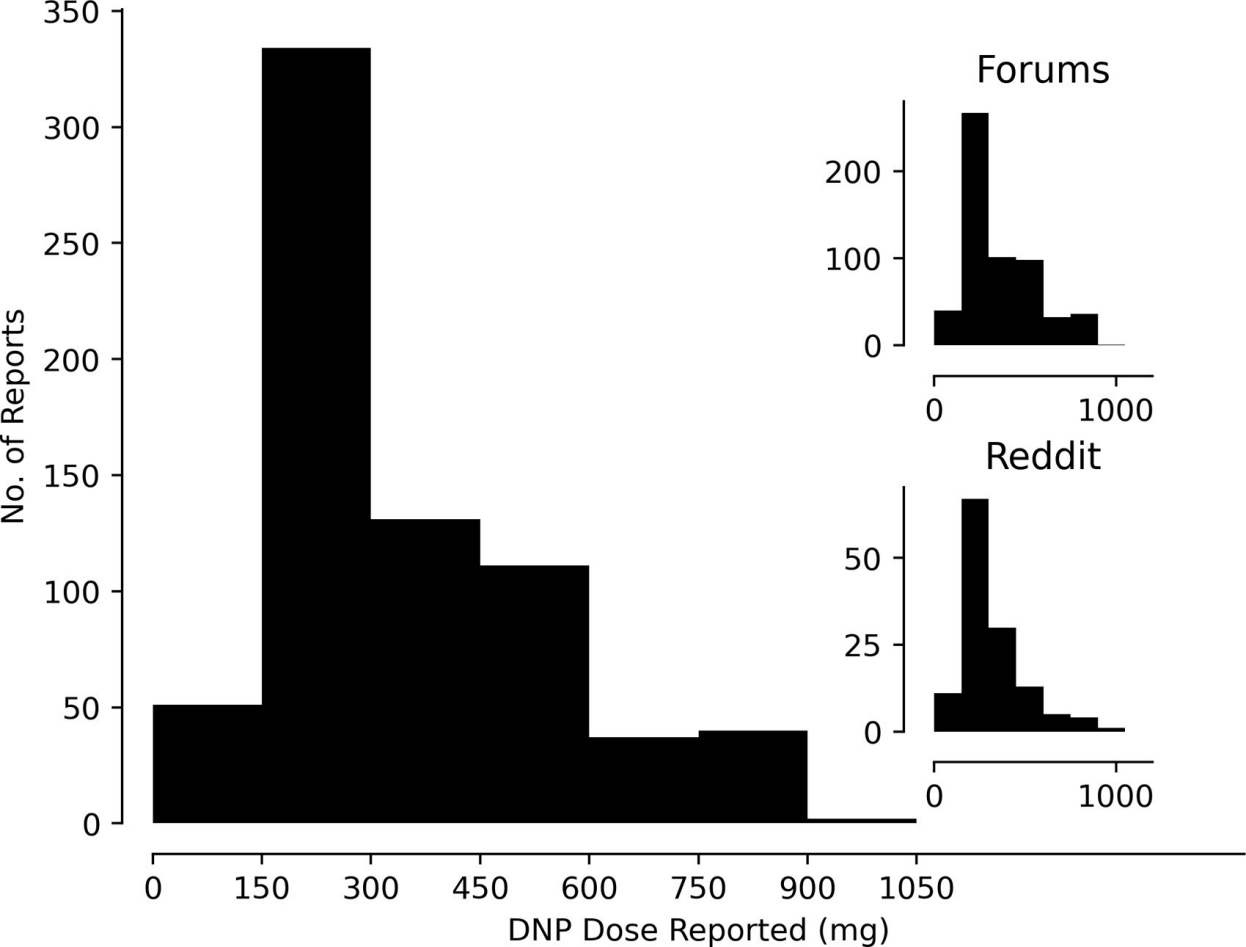
Distribution of doses mentioned for a single DNP ingestion. X-axis, dose (mg). Y-axis, number of posts mentioning each dose. Bin size 150 mg. Insets show distribution of individual sources, (online web) forums (right top) and Reddit (right bottom). X- and Y-axes of insets and main figure use the same units.

**Fig 3:**
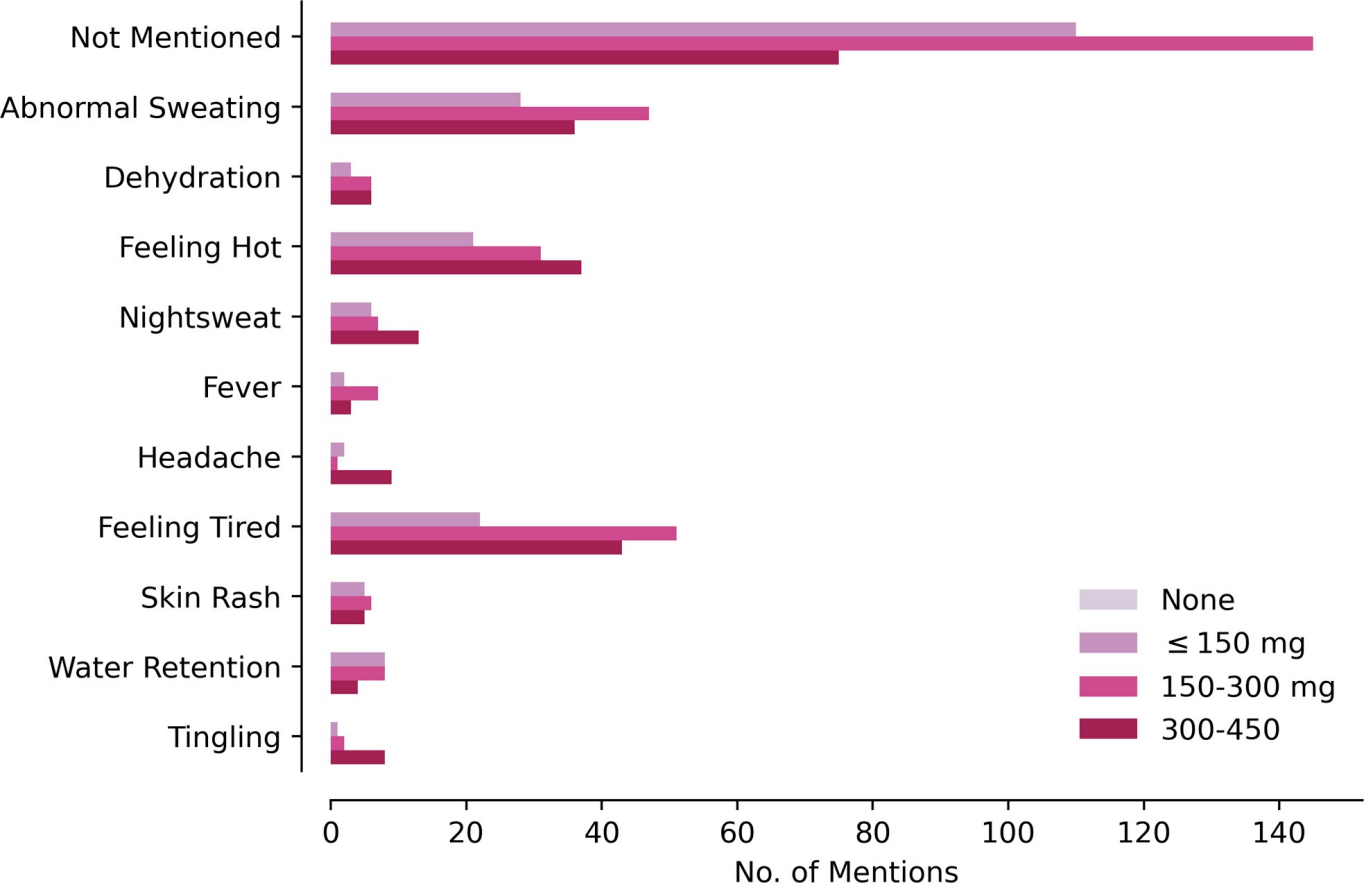
Reported effects of DNP. Y-axis, reported dose in mg. X-axis, number of mentions. Color of bar indicates dosage.

In the web forums, the most commonly mentioned doses were 150 mg twice a day (40 out of 105 posts, 38%) and 300 mg three times a day (25 out of 105 posts, 23.8%), leading to total daily doses of 300 mg and 600 mg, respectively. In r/DNP, the most mentioned doses were 200 mg once a day (19/68, 28%) and 300 mg once a day (16/68, 24%). **Fig 2** shows the distributions of reported doses. We chose bins of 150 mg to reflect the average weight of substances ingested in one DNP pill. The most mentioned effects were increased sweating, fatigue, and parestheisas (**Fig 3**). The most common effect categories were fatigue and thermoregulatory (**Table 2)**. r/DNP posts mentioned tingling, paresthesias and concern about peripheral neuropathy (35/969, 3.6%). No posts from the other online forums mentioned this concern. The effects were not uniformly distributed across dosages (χ^2^-test, p < 0.001). Reports of thermoregulatory (sweating, feeling hot flashes or flushed) and fatigue-related symptoms were statistically significantly more frequent at reported daily doses greate than 150 mg than doses below 150 mg, but not more frequent between reported daily doses of 300 to 450 mg than 150 to 300 mg (post-hoc χ^2^-test with Bonferroni correction). Posts discussing lower doses were less likely to mention any adverse effect.

**Table 2:** Effects of DNP grouped by category. All doses in mg. Bold numbers indicate row and column sums. * denotes row-wise p-value not calculated because of too small numbers. Otherwise, p-values calculated by the chi-square text. Bold text denotes p-value < 0.05 after Bonferroni correction with factor of 4. If any cell less than 4, the Freeman-Halton extension of Fisher’s exact test was used instead of the chi-squared test.

		Dose Mentioned					
		< 150	150 to 300	300 to 450	None	**Total**	**p-value**
Nature of Effect	**Fatigue**	**22**	**51**	**43**	**6**	**122**	**0.04**
	Dehydration	11	14	10	1	36	0.86
	**Neurologic**	**3**	**3**	**17**	**2**	**25**	**< 0.01**
	Dermatologic	5	6	5	0	16	*
	**Thermoregulatory**	**57**	**92**	**89**	**12**	**250**	**0.04**
	**Not Mentioned**	**110**	**145**	**75**	**52**	**382**	**0.01**
	**Total**	208	311	239	73	831	

### Coingestants

**Fig 4:**
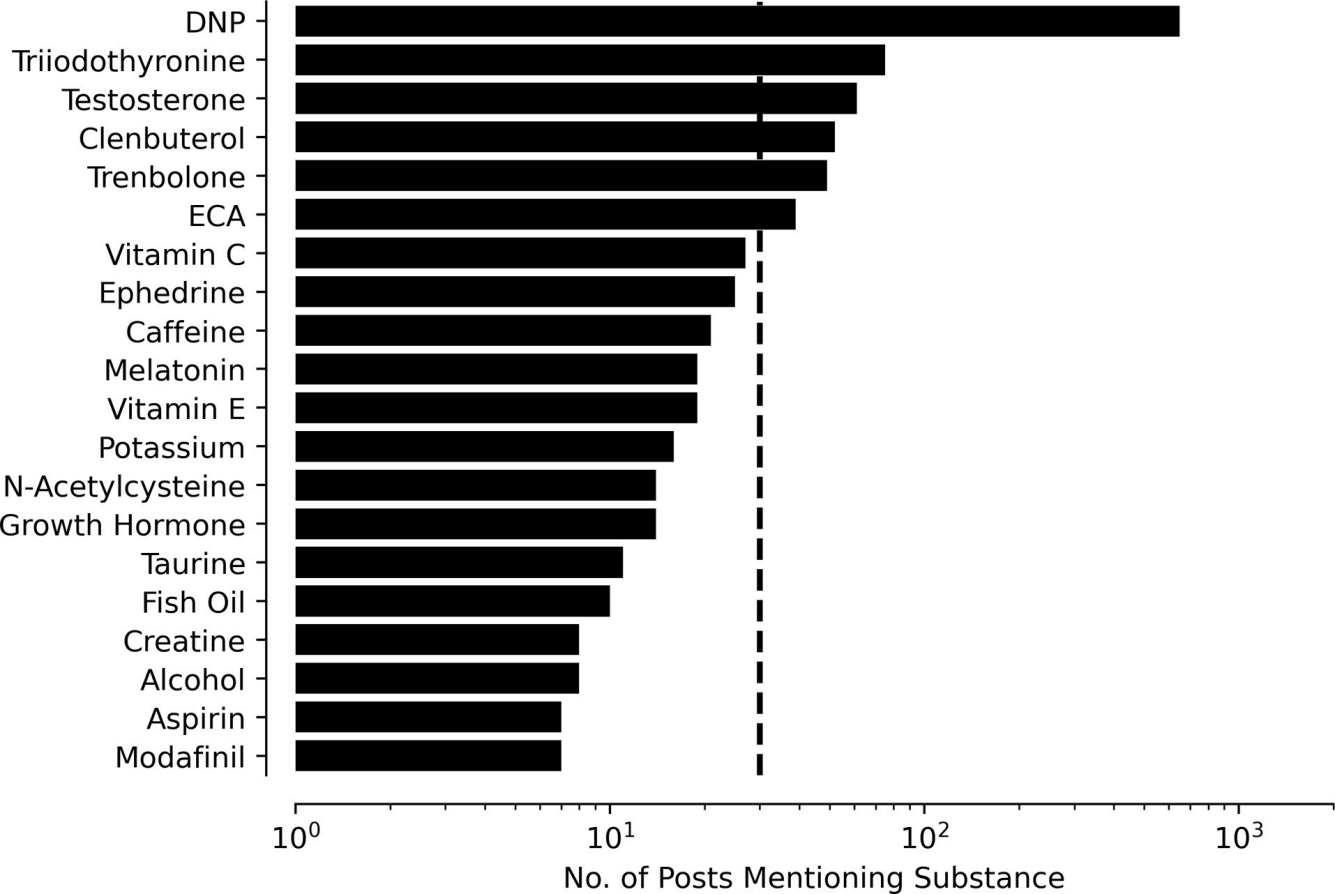
Distribution of substance mentions over all unique posts. Y-axis indicates name of substance; IUPAC or generic name used when possible. X-axis indicates number of posts in which substance was mentioned on log scale. Horizontal dashed line indicates threshold for statistical significance after Benjamini-Hochberg correction.

Of the 661 posts in the web forums, 140 (21.2%) mentioned at least one substance (66 total unique substances) besides DNP. In r/DNP, 266 posts (40%) mentioned an additional substance (153 total unique substances). **Fig 4** shows the frequency of comentions of substances with DNP across web forums and r/DNP. The vertical dashed line denotes the threshold for a statistically significant comention. Triiodothyronine, testosterone, clenbuterol, trenbolone, and ECA were significantly comentioned with DNP. Triidothyrodine is the biologically active form of thyroid hormone. ECA refers to the combination of ephedrine, caffeine, and aspirin. Other substances displayed in **Fig 4** relate to anabolism (taurine, growth hormone, methylstenbolone, deca [nandrolone]), substances used to offset the unwanted effects of steroid use (clomiphene, fish oil, vitamin E, vitamin C), and substances perceived to increase weight loss (albuterol, ephedrine, aspirin).

## Discussion

The goal of this study was to understand sublethal toxicity from DNP using online self-reporting. We found that the most mentioned daily dose was 300 mg and the most mentioned adverse effects were related to fatigue, increased body temperature, and paresthesias. Contributors to r/DNP mentioned paresthesias and a concern for peripheral neuropathy. The only neurological issue the web forums mentioned was a headache. The most co-mentioned substances were related to androgenic anabolic steroids or other weight loss agents.

We analyzed unstructured text from social media because no other data stream is available for sublethal effects. DNP is banned for human consumption by the FDA. No equipoise for clinical trials exists because there is no known safe dose in humans. Logs from Poison Control Centers and autopsy records bias towards more severe presentations.

Our study agrees with prior smaller qualitative analyses of DNP users on social media who described adverse effects of fatigue and profuse sweating, and a daily dose of 325 mg [13]. Side-effects were more frequently mentioned at doses of 150–300 mg than at doses less than 150 mg, suggesting a dose-response effect that supports the validity of this approach and complements case reports from the toxicological literature [23]. However, there were also more comments at 150–300 mg than at higher, suggesting reporting bias and raising the possibility that we underestimated the prevalence of toxicity at doses above 150–300 mg.

### Limitations

We did not analytically confirm the amounts users reported ingesting. Only 3 posts discussed sending a sample to a laboratory to verify composition. None of those posts discussed the results of those analyses.

Posts did not consistently distinguish between crystalline and powder forms. The crystalline form is said to contain 75% DNP by weight and is sold in 100 mg aliquots. The powdered form is said to contain 100% DNP and is sold in 150 mg aliquots. Users described measuring the powder out, but they did not specify whether the weight referred to the total weight of the powder or amount of DNP. The accuracy of commercial scales can vary from the order of 1 mg to 1 g. Posts rarely described how users ingested DNP. At room temperature DNP is a powder. Anecdotally, DNP is consumed orally. It could be insufflated or injected. Injection would likely lead to an overwhelming infection and rapid death.

Users did not specify how they acquired DNP. Those who acquire DNP from pesticide vendors may have a consistent source of DNP, but a source not fit for human consumption. The amount of DNP in products sold ‘as is’ for ‘research purposes’ may vary substantially.

Users mentioned taking multiple substances in addition to DNP. Our data were only adequate to determine the statistical significance of the top 5 most frequently co-mentioned substances. Larger studies may have enough power to identify more substances. We did not consider the time frame of co-use. Using two substances simultaneously can have different effects than taking two substances hours, days, or weeks apart. Our method cannot detect polydrug use that was not reported online.

A unique strength and limitation of online sources is the anonymity of the user. Anonymity may facilitate discussion on illegal or sensitive topics. But it limits our insight into the demographics of the population under study and our ability to confirm the assertions users make. We excluded duplicate posts by the same user, posts with the exact same text posted from two different users, and excluded posts that offered to sell DNP. It is, nevertheless, still possible that some posts arise from non-human posters (“bots”). We do not know if users were describing observed or anticipated effects. All the comments were judged as plausible by two domain experts. Neurological effects of DNP have not been reported in the clinical literature. Organochlorine pesticides, which are structurally similar to DNP, are associated with neurotoxicity, but current research suggests this reflects dysregulation of cholinergic signaling [24]. Mitochondrial function is necessary for the maintenance of myelin [25].

The mention of paresthesias in r/DNP but not web forums suggests that content varies between online sources. In r/DNP 35 posts discussed neurotoxicity, 14 of which mentioned a dose, yielding too few data to draw any statistically valid conclusions. Future work could compare the variation in content on the same topic across online sources.

## Conclusions

2,4-dinitrophenol (DNP) is an alluringly potent proposed weight loss agent. Our work here suggests that doses greater than 150 mg each day are associated with more signs of hyperthermia (abnormal sweating, elevated temperature, sensation of dehydration), fatigue, and possibly nerve injury than doses less than 150 mg. This investigation demonstrates the utility of social media in providing plausible preliminary data on substance usage that would be challenging to obtain by other methods.
